# The histone deacetylase HDA19 controls root cell elongation and modulates a subset of phosphate starvation responses in *Arabidopsis*

**DOI:** 10.1038/srep15708

**Published:** 2015-10-28

**Authors:** Chun-Ying Chen, Keqiang Wu, Wolfgang Schmidt

**Affiliations:** 1 Molecular and Biological Agricultural Sciences Program, Taiwan International Graduate Program, Academia Sinica, Taipei, Taiwan, and National Chung-Hsing University, Taichung, Taiwan; 2 Graduate Institute of Biotechnology, National Chung-Hsing University, Taichung, Taiwan; 3 Institute of Plant and Microbial Biology, Academia Sinica, Taipei, Taiwan; 4 Institute of Plant Biology, National Taiwan University, Taipei, Taiwan; 5 Biotechnology Center, National Chung-Hsing University, Taichung, Taiwan; 6 Genome and Systems Biology Degree Program, College of Life Science, National Taiwan University, Taipei, Taiwan

## Abstract

The length of root epidermal cells and their patterning into files of hair-bearing and non-hair cells are genetically determined but respond with high plasticity to environmental cues. Limited phyto-availability of the essential mineral nutrient phosphate (Pi) increases the number of root hairs by longitudinal shortening of epidermal cells and by reprogramming the fate of cells in positions normally occupied by non-hair cells. Through analysis of the root morphology and transcriptional profiles from transgenic *Arabidopsis* lines with altered expression of the histone deacetylase *HDA19*, we show that in an intricate interplay of Pi availability and intrinsic factors, HDA19 controls the epidermal cell length, probably by altering the positional bias that dictates epidermal patterning. In addition, HDA19 regulates several Pi-responsive genes that encode proteins with important regulatory or metabolic roles in the acclimation to Pi deficiency. In particular, HDA19 affects genes encoding SPX (SYG1/Pho81/XPR) domain-containing proteins and genes involved in membrane lipid remodeling, a key response to Pi starvation that increases the free Pi in plants. Our data add a novel, non-transcriptionally regulated component of the Pi signaling network and emphasize the importance of reversible post-translational histone modification for the integration of external signals into intrinsic developmental and metabolic programs.

The formation of root hairs, tip-growing unicellular extensions of root epidermal cells emerging from specialized cells referred to as trichoblasts, is a well-explored model for the specification and differentiation of plant cells. In *Arabidopsis* and other members of the Brassicales, root hairs are arranged in a striped pattern of longitudinal files of hair and non-hair cells that is controlled by a sophisticated mechanism in which cell-to-cell-movement of transcriptional regulators and extracellular signaling pathways regulate the allocation of the alternating binary cell fate^1^,^2^,^3^. A positional signal, probably induced by JKD in the root cortex^4^, is perceived by the leucine-rich repeat receptor kinase SCM localized on the plasma membrane of epidermal cells^5^,^6^. The signal is supposedly more abundant in epidermal cells that contact an anticlinal wall of underlying cortical cells (H position) owing to the larger surface contact area relative to cells that are located over periclinal cortical cells (N position). This bias derived from cortical cells is amplified by a feedback mechanism that dictates higher SCM abundance in H positions relative to cells in N positions^7^. SCM suppresses the non-hair transcription factor WER, which leads to higher WER abundance in non-hair cells relative to hair cells. Together with the bHLH MYC transcription factor GL3 and the WD repeat protein TTG1, WER forms an activator complex that promotes the non-hair cell fate by supporting the transcription of the homeodomain protein GL2^8^. The activator complex also induces the expression of the R3 MYB transcription factor CPC, a positive regulator of the hair fate, which migrates via plasmodesmata to hair cells where it competes with WER for binding to the activator complex^9^. A higher CPC/WER ratio compromises the function of the activator complex and cells enter the hair cell fate. GL3 is preferentially expressed in hair cells and moves to N-positioned cells to reinforce the non-hair cell fate.

Root hair formation is highly responsive to environment signals. For example, low phyto-availability of phosphate (Pi) alters root epidermal cell differentiation by inducing a nutrient-specific program, which includes the suppression of primary root growth by root apical meristem exhaustion and the restricted elongation of root cells. In addition, the elongation time of root hairs is prolonged and their growth rate is enhanced, leading to a significant increase in root hair length^10^. The higher root hair frequency of Pi-deficient plants results from reduced longitudinal elongation of epidermal cells and additional hair fate assignment of N-positioned cells^11^. This response has been explored for the widely used Columbia (Col-0) strain, but large variations appear to exist across *Arabidopsis* accessions^12^.

A plethora of genes is differentially expressed between Pi-deficient and Pi-replete plants^13^,^14^,^15^. Co-expression analysis revealed that large modules of Pi-responsive genes are associated with induction of the root hair phenotype typical of Pi-deficient plants^16^. However, not all of the genes that determine this phenotype showed altered expression in response to changing Pi supply. A forward genetic screen identified the ubiquitin-specific protease UBP14 (PER1) as being essential for root hair elongation specifically at low Pi supply^17^. A similar screen established the homeodomain protein ALF6 (PER2) as a critical regulator of the Pi-deficiency phenotype^18^. ALF6 binds to trimethylated lysine 4 of histone 3 (H3K4me3) and is thought to affect the transcript maturation and stability of downstream targets^18^,^19^. Interestingly, other post-translational modifications (PTMs) of histones such as deacetylation of lysine residues by the histone deacetylase HDA18 are important for root hair patterning under Pi-replete conditions. Alterations in the activity of HDA18 lead cells in N positions to adopt the hair cell fate^20^,^21^.

Class I HDAs such as HDA6 and HDA19 were reported to participate in several biotic and abiotic stress responses and play regulatory roles in developmental programs^22^. Here, we set out to explore the role of the RPD3/HDA1 superfamily member HDA19 on the translation of the Pi deficiency signal into the phenotype typical of Pi-deficient plants. We report that HDA19 controls several responses to Pi starvation, including root hair density, epidermal cell elongation and membrane lipid remodeling, thereby accentuating the critical involvement of chromatin modifications in phenotypic plasticity.

## Results

### Mutations in Class I HDAs compromise the Pi deficiency-induced increase in root hair density

**Figure 1:**
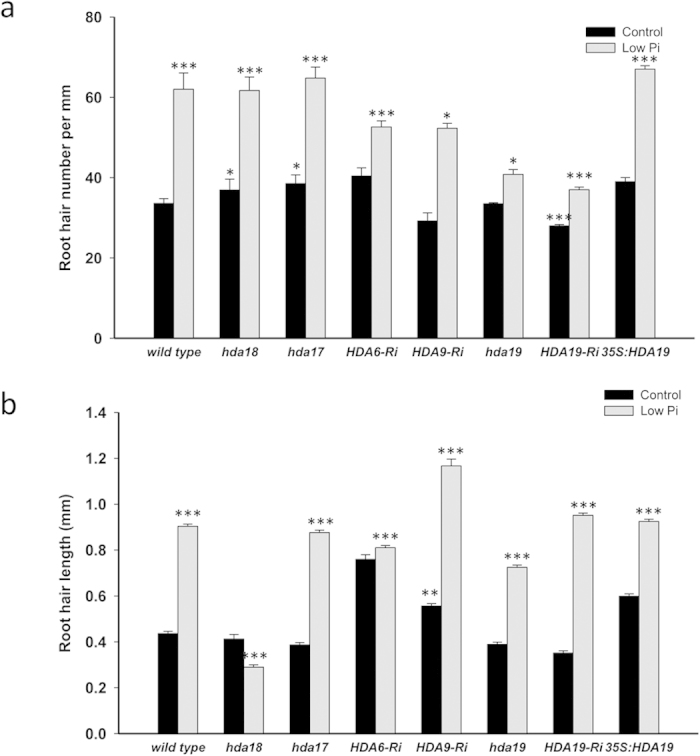
Effect of low Pi conditions on the root hair formation of lines with altered HDA abundance grown under Pi-replete (control) and low Pi conditions. (**a**) Root hair number. (**b**) Root hair length. Seedlings were grown on control and low Pi media for 14 days. Data represent means of ten plants for each genotype and growth type ± SE. The asterisks denote statistically significant differences to Pi-replete wild-type plants based on Student’s t-test (*P < 0.05, **P < 0.01, ***P < 0.001). HDA19-Ri, HDA19-RNAi.

Pi deficiency affects the development of epidermal cells in a nutrient-specific manner, resulting in an increase in both length and density of root hairs^23^,^24^. The molecular mechanism governing these changes remains elusive. Root hair patterning under control (Pi-replete) conditions is dependent on correct acetylation of the N-terminal tails of core histones^20^,^21^. To investigate whether reversible histone acetylation is also important for the Pi deficiency-induced root hair phenotype, we tested homozygous mutants or knock-down lines defective in the expression of *HDA18* (class II of the RPD3/HDA1 superfamily), *HDA17*, an unclassified member of the RPD3-like superfamily, and the class I members *HDA6*, *HDA9* and *HDA19* for their root hair phenotypes under control and low Pi conditions. All lines showed a significant increase in root hair density when grown on low Pi media. This increase was less pronounced in all three class I HDAs, which was in particular evident in the *hda19* mutant (Fig. 1a). HDA19-RNAi plants showed a response similar to that of the *hda19* mutant. Transgenic plants over-expressing *HDA19* driven by the 35S promoter (35S:HDA19) produced more root hairs under low Pi conditions when compared to the wild type (Fig. 1). Notably, HDA19-RNAi formed fewer root hairs than the wild type also under control conditions and mutants defective in the expression of *HDA18* and *HDA17* produced significantly more root hairs than the wild type under Pi-replete conditions.

Root hair length was increased by more than two-fold upon Pi starvation; a Pi deficiency-induced increase was observed for all lines except for *hda18* (Fig. 1b). Under Pi-replete conditions, only for HDA9-RNAi plants a deviation in root hair length from that of the wild type was observed.

### HDA19 activity controls epidermal cell length

**Figure 2:**
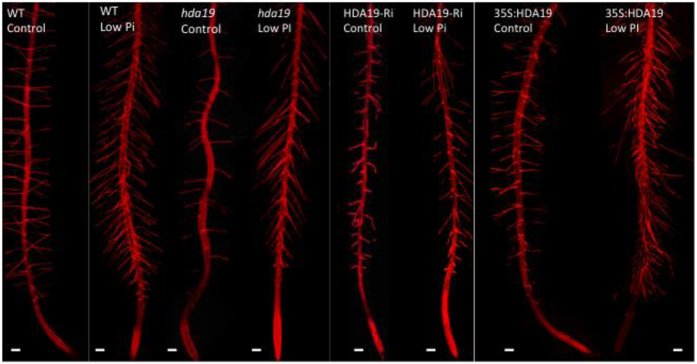
Root hair phenotypes of the investigated lines grown under Pi-replete (control) and low Pi conditions. Pictures show compiled confocal images. Scale bar = 100 μM.

**Figure 3:**
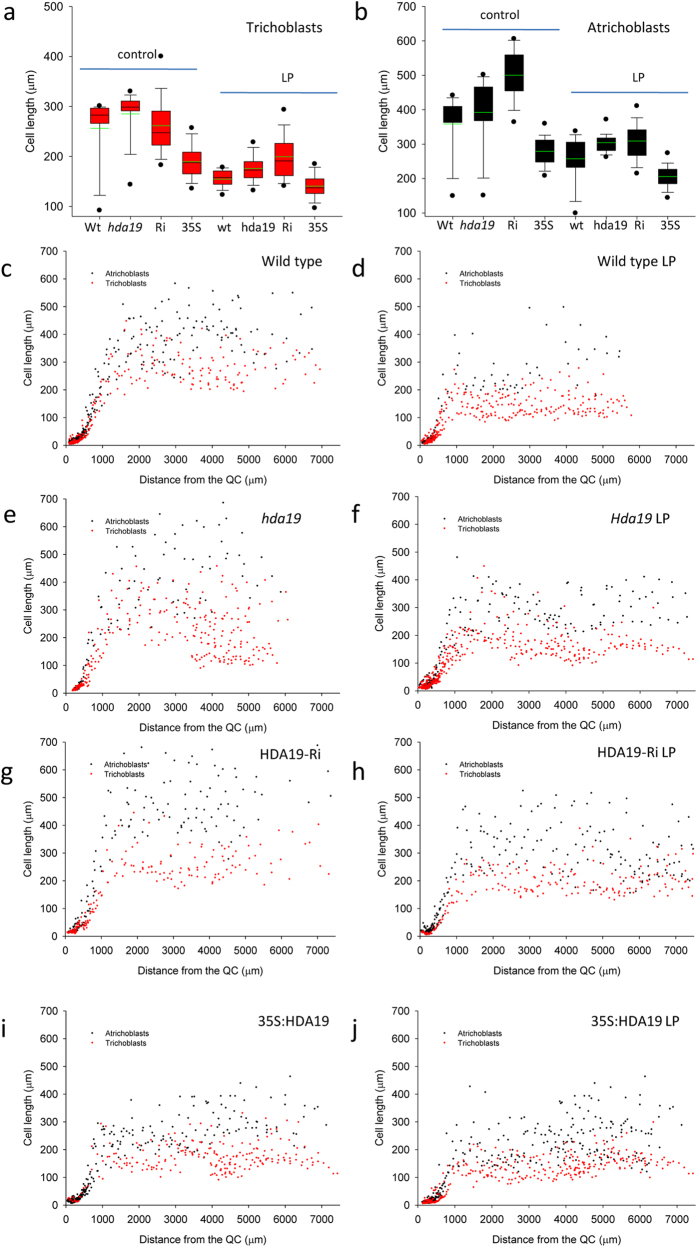
Longitudinal length of epidermal cells. (**a,b**) Length of trichoblasts (**a**) and atrichoblasts (**b**) of wild type (wt), *hda19*, HDA19-Ri (Ri) and 35S:HDA19 (35S) plants under control (**c,e,g,i**) and low phosphate (LP; **d,f,h,j**) conditions. (**c–j**) Data in a and b show box plots of the average of three roots per genotype and treatment (on average 550 cells per growth- and genotype). The mean is shown in red. Data shown in (**c-j**) are from the analysis of compiled confocal micrographs from three different roots per treatment and genotype. QC, quiescent center.

In *Arabidopsis*, the driving force for the increase in root hair density upon Pi deficiency is a diminished longitudinal elongation of trichoblasts^11^. To investigate whether the change in the frequency of root hairs caused by altered *HDA19* expression is a consequence of altered trichoblast length, we analyzed the length of epidermal cells of the genotypes under investigation. To this end, we compiled micrographs of individual roots comprising the first six to eight millimeter of the root tip and measured the length of the cells in relation to their distance from the quiescent center. This procedure was repeated with three different roots for each genotype (wild type, HDA19-RNAi and 35S:HDA19) and growth type (Pi-replete and low Pi). The respective root hair phenotypes are shown in Fig. 2. Cell length measurements revealed that for both growth types HDA19 abundance is critical in controlling the length of both trichoblasts and atrichoblasts (Fig. 3). While knock-down lines produced markedly longer cells than the wild type, short epidermal cells were observed in 35S:HDA19 plants. HDA19-RNAi plants showed a large variation in cell length under Pi-replete conditions, which were on average comparable to those of the wild type. The distribution of the cell lengths along the roots is shown in Fig. 3c–j.

### Overexpression of *HDA19* induces additional hair cell fate assignment in Pi-deficient plants

**Figure 4:**
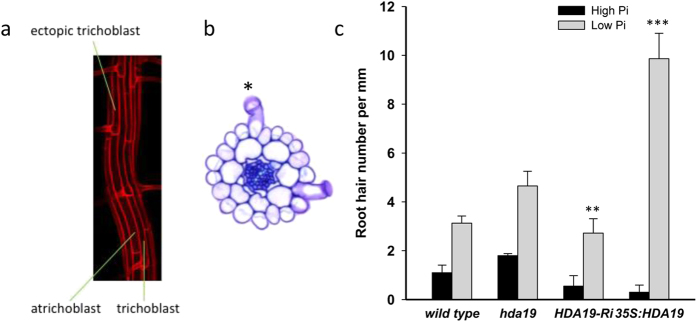
Formation of ectopic root hairs. (**a**) Confocal micrograph showing a trichoblast in an atrichoblast cell fate in a Pi-deficient root. (**b**) Cross-section showing the formation of root hairs in the H and N position (*). (**c**) Quantification of root hairs in N position based on the analysis of cross-sections. Seedlings were grown on control and low Pi media for 14 days. Data represent means of ca. 40 cross-section from 10 plants for each genotype and growth type ± SE. Asterisks denote statistically significant differences to Pi-replete wild-type plants based on Student’s t-test (*P < 0.05, **P < 0.01, ***P < 0.001).

In addition to a decrease in cell length and, associated with this response, an increase in root hair density in the H position, growth on Pi-depleted media increases the probability of epidermal cells in the N position to adopt the H cell fate^24^. To investigate whether the increase in root hair density in 35S:HDA19 plants in response to Pi starvation is solely due to shortening of epidermal cells or whether additional H cell fate acquisition contributes to the phenotype, we conducted a detailed analysis of the frequency of root hairs in ectopic positions based on cross-sections. This analysis revealed a dramatic (33-fold) increase in the number of ectopic roots in 35S:HDA19 plants when grown on low Pi media. Although this massive increase was due to the very low number of ectopic roots in this genotype under control conditions (0.3 *vs* 1.1 in the wild type), the frequency of ectopic root hairs formed under low Pi conditions was still three times higher in the 35S:HDA19 line when compared to that of the wild type (Fig. 4), indicative of a strongly increased probability to form hairs in ectopic positions.

### *HDA19* abundance affects leaf responses to Pi starvation

**Figure 5:**
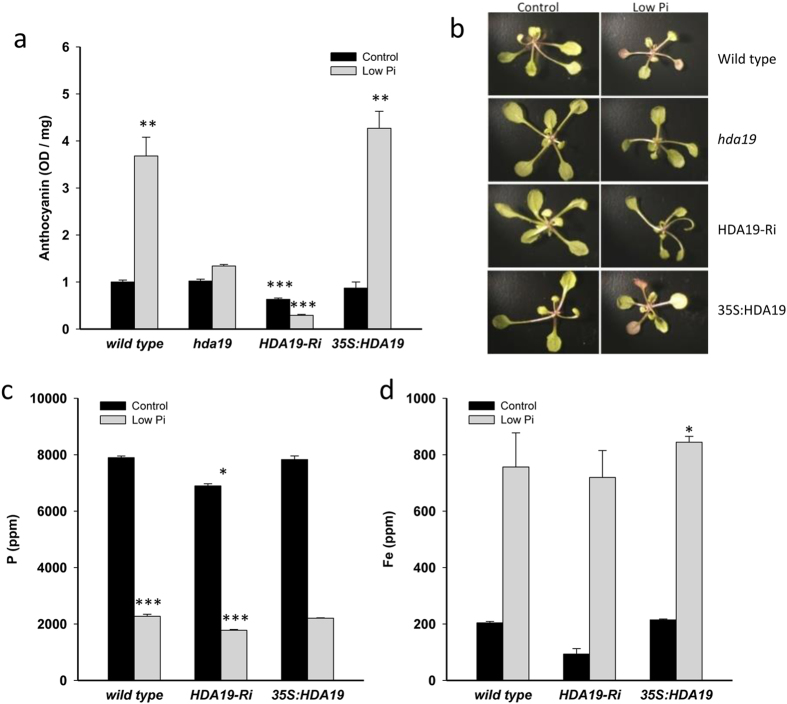
Anthocyanin production, phosphorus and iron concentration in leaves of transgenic lines with altered *HDA19* abundance. (**a**) Anthocyanin levels normalized to Pi-replete wild-type plants. (**b**) Leaves of the investigated lines under control and low Pi conditions. (**c**) Phosphorus concentrations in leaves and (**d**) iron concentrations in roots as determined by ICP-OES. Seedlings were grown on control and low Pi media for 14 days. Data represent means of ten plants for each genotype and growth type ± SE. Asterisks denote statistically significant differences to Pi-replete wild-type plants based on Student’s t-test (*P < 0.05, **P < 0.01, ***P < 0.001).

A further hallmark of Pi-deficient plants is an accumulation of anthocyanin in leaves to protect nucleic acids and chloroplasts from photo-oxidative damage due to limited photosynthesis. In wild-type plants, a *circa* 4-fold increase in anthocyanin concentration was observed after transfer of the plants to low Pi media (Fig. 5a,b). Pi-deficient *hda19* mutants showed a very small increase in anthocyanin levels over Pi-replete plants; in Pi-deficient HDA19-RNAi plants anthocyanin levels were reduced relative to the wild type when grown on Pi-replete or on low Pi media. Anthocyanin concentrations in 35S:HDA19 plants did not differ from that of the wild type (Fig. 5a,b). Since anthocyanin synthesis is controlled by R3 and R2R3 MYB transcription factors, which also regulate epidermal cell fate (*i.e.* CPC and GL3), it was tempting to assume that HDA19 abundance altered root hair density by altering their expression. However, transcript abundance of *CPC* and *GL3* did not differ significantly across the genotypes under investigation.

Determination of leaf Pi concentrations by ICP-OES did not reveal significant differences between the wild type and 35S:HDA19 plants under both growth conditions. In HDA19-RNAi plants, the Pi concentrations in leaves was significantly decreased under control conditions, but no changes were observed when plants were grown on low Pi media (Fig. 5c). Pi concentrations in roots did not differ between the genotypes. Iron accumulates in Pi-deficient plants^13^ and may interfere with cell elongation. As anticipated from previous studies, the concentration of iron was dramatically increased under Pi-deficient conditions but did not vary much among the genotypes, except a significant increase in roots of 35S:HDA19 plants grown on low Pi media (Fig. 5d). No differences in leaf iron concentration were observed between the genotypes under investigation.

### HDA19 modulate the expression of Pi-responsive genes

To understand the molecular events that determine the phenotypes of lines with altered *HDA19* expression, we conducted transcriptional profiling experiments of HDA19-RNAi and 35S:HDA19 plants grown under control and low Pi conditions. A subset of 86 Pi-responsive genes was expressed two-fold lower in HDA19-RNAi plants when compared to 35S:HDA19 lines (Supplemental Table 1). Among the genes with the highest difference was *SPX3* that showed a 17-fold difference between the two genotypes. *SPX1*, which encodes a key regulator of the Pi starvation response, and another SPX (SYG1/Pho81/XPR) domain protein, At2g38920, were less Pi-responsive in HDA19-RNAi lines. Furthermore, *MGD3*, a key enzyme in galactolipid biosynthesis, and the purple acid phosphatase *PAP23* were down-regulated in HDA19-RNAi plants. Galactolipid biosynthesis is induced by Pi deficiency as part of metabolic pathway during which phospholipids (PLs) in membranes are replaced with the galactolipid digalactosyldiacylglycerol and the sulfolipid sulfoquinovosyldiacylglycerol (‘membrane lipid remodeling’^25^). Excretion of PAPs aimed at re-mobilizing Pi represents a further key PSR response. *PAP23* is co-expressed with *SPX1*/*SPX3* and genes involved in galactolipid biosynthesis that were strongly induced upon Pi starvation such as *MGD3*. The phospholipase D *PLDζ2* and the glycerophosphodiester phosphodiesterase *GDPD6* showed reduced expression in the *HDA19* knockdown line. Also PLDζ2 and GDPD6 have been associated with the hydrolysis of PLs as part of membrane lipid remodeling^26^,^27^. Notably, induction of *PLDζ2* upon Pi starvation was dependent on functional CPC, a positive regulator of the root hair cell fate^28^. GDPDs convert lysophosphatidylcholine derived from PLs to glycerol-3-phosphate, a precursor of galactolipids. *CAR8*, another repressed gene in the HDA19-RNAi line, is tightly co-expressed with genes involved in galactolipid biosynthesis. Five genes with reduced expression in HDA19-RNAi plants encode protein kinases (*CRK24*, *CRK6*, *CRK45*, *HT1*, At2g17170), indicating a possible post-transcriptional regulation of the cell length. Also of note, two genes involved in cell-to-cell signaling, *RALFL9* and *RALFL27*, members of the peptide family with similarity to tobacco Rapid Alkalinization Factor (RALF), were lower expressed in HDA19-RNAi relative to 35S:HDA19 plants.

Consistent with a role of HDA19 as a gene repressor, slightly more genes (106) showed higher induction upon Pi starvation in HDA19-RNAi relative to 35S:HDA19 plants. The most pronounced differences were observed for several genes encoding unknown proteins (Supplemental Table 2). Interestingly, 11 genes in this suite (*IGMT2*, *GSTL1*, At3g01260, *NMT3*, *SKOR*, *GSTF3*, At5g22555, At2g43590, At4g01430, At4g19370 and *FRD3*) were previously defined as Fe-responsive^29^, while only one such gene (*NAS2*) was among the genes that showed lower expression in the HDA19-RNAi line. About half of the genes in this group were up-regulated upon iron deficiency and the other half showed reduced expression in iron-deficient plants, suggesting that a lack of functional HDA19 compromises cellular iron homeostasis. Consistent with a role in cell differentiation, several genes (20 out of 106) encode proteins that are predicted to be located in the extracellular region. Three genes (At5g48540, At1g07560 and *ATPI4Kγ3*) encode protein kinases.

**Figure 6:**
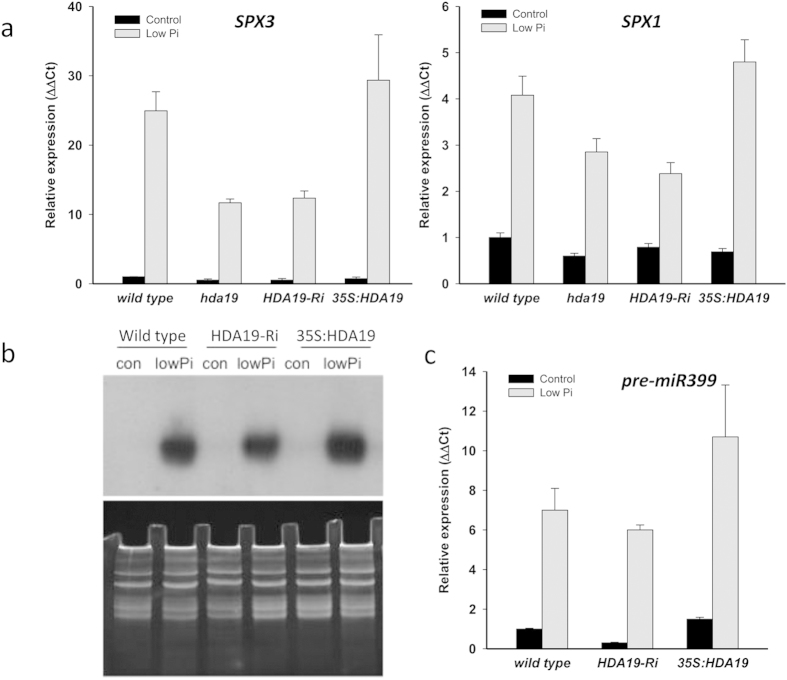
Effect of altered *HDA19* expression on Pi-homeostasis genes. (**a**) Expression analysis of *SPX3* and *SPX1* by qRT-PCR. (**b**) Northern blot analysis of mature *miR399d* abundance. (**c**) qRT-PCR analysis of *pri-miR399d* expression. Seedlings were grown on control and low Pi media for 14 days. Values are means ± SE from three biological replicates.

To validate the observed changes in gene expression, we determined the transcript levels of selected genes by qRT-PCR. In roots of Ws-2, the wild type of the transgenic lines, *SPX3* transcripts were dramatically increased by Pi deficiency. To allow comparison with the widely used Col-0 accession, we also determined the expression of *SPX3* in Col-0 roots, which showed a similar increase in transcript abundance. In both growth types of the *HDA19* knock-down lines, *SPX3* transcript levels were markedly reduced relative to the wild types; no changes were observed for the 35S:HDA19 line (Fig. 6). A similar picture was observed for *SPX1*, albeit with less pronounced changes.

miRNA399 is a systemic key regulator of the PSR. To determine whether systemic Pi signaling is affected in lines with altered *HDA19* expression, we measured the abundance of mature miR399d and its primary transcript (pri-miR399d) by Northern blotting and qRT-PCR, respectively. Northern blot analysis of miR399d revealed a massive increase in RNA abundance upon Pi deficiency with a slight reduction in the HDA19-RNAi line and a more pronounced band for roots of the 35S:HDA19 line (Fig. 6a). Expression analysis of pri-miR399d supported this pattern. In the wild type, the abundance of pri-miR399d was circa 7-fold increased (Fig. 6b). Pri-miR399d expression was not significantly altered in the knock-down line; although, a trend of reduced expression was noted. In 35S:HDA19 plants, a marked increase in pri-miR399d transcript levels was observed in roots from plants grown under both Pi-replete and low Pi conditions (Fig. 6b), indicative of higher miR399d activity in 35S:HDA19 plants. It thus appears that HDA19 is critical in systemic Pi signaling.

## Discussion

Reversible modification of core histones is an important regulator of gene activity, modulating DNA-templated processes such as transcription and repair. Acetylation of the ε-amino group of specific lysine residues neutralizes the positive charge of the histone tails, thereby weakening the interaction with the DNA and increasing the accessibility of the DNA for transcriptional regulators^30^. Histone acetylation is generally associated with gene activation. However, histone PTMs are not ultimately dictating gene expression and the interpretation of histone marks has been described as translation of a ‘histone language’ rather than deciphering a rigid code^31^. Thus, although HDAs are generally defined as transcriptional repressors, decreased acetylation is not a mandatory partner of gene repression. This is supported by the fact that in the present investigation the number of Pi-responsive genes that were greater than two-fold increased in HDA19-RNAi relative to 35S:HDA plants were only slightly higher than genes with two-fold lower expression in the knock-down line. The phenotypic changes observed in the transgenic lines may thus be caused by both activation and repression of genes by altered HDA19 abundance.

### HDA19 modulates cell-to-cell signaling

The longitudinal length of epidermal cells is controlled by a yet unidentified signal that presumably originates in the root cortex. Mutants that cannot sense or transduce this signal such as *scm* or *wer* produce short cells characteristic of root hair-forming cells^11^. Mathematical modeling suggested that lack of perception or reduced strength of the positional signal delays the time point at which non-hair cells exit the default trichoblast pathway, resulting in short, trichoblast-like epidermal cells^11^. It was further suggested that the cortical bias is reduced in Pi-deficient plants, leading to a reduction in epidermal cell length and reduced rigidity of epidermal patterning. The current data imply that HDA19 is modulating this signal, causing opposite cell length in knock-down and 35S:HDA19 lines. HDA19 might either affect the generation of the positional signal, its perception or regulate signaling nodes that relay the cortical bias to down-stream target genes. A weaker signal defines a less strict pattern with a higher probability of epidermal cells entering the (default) hair fate, leading to an appreciable increase in ectopic root hairs in 35S:HDA19 lines when grown on low Pi media. This scenario is supported by the observation that compromising the positional signal by separating the epidermal cell layer from the cortex in Brassicaceae caused all epidermal cells to enter the hair cell fate^32^.

Ethylene is an important modulator of both root hair morphogenesis and cell elongation^33^,^34^. Ethylene affects root hair initiation downstream of RHD6 and does not interfere with the early transcriptional regulators that dictate the fate of the cells^34^,^35^,^36^. HDA19 has been previously implicated in ethylene signaling^37^, and it may be assumed that HDA19 affects ethylene production or signaling, which in turn alters the root hair phenotype in lines with altered *HDA19* expression. While we cannot exclude that HDA19 exerts its effect via ethylene signaling, our results are more in line with an altered strength of a cortical signal. Ethylene induces long root hairs, which was not observed in the 35S:HDA19 line. Also, ethylene induces the formation of ectopic root hairs, which was only observed when plants were grown on low Pi medium. Moreover, ethylene-related genes that were previously found to be up-regulated in leaves of 35S:HDA19 lines such as *ERF1*^37^ did not differ in expression between 35S:HDA19 and HDA19-RNAi roots, further arguing against the assumption that HDA19 chiefly acts through ethylene signaling on the root phenotype. Together, the data can be more readily explained by modified strength or perception of a positional signal in transgenic lines with altered expression of *HDA19*.

### Histone modifications may interact to regulate responses to Pi starvation

Previously, we identified the PHD finger protein ALF6 in a forward genetic screen as a critical player for root hair elongation under Pi-deficient conditions^18^. ALF6 is a *bona fide* histone reader that binds to H3K4me3^38^. Beside compromised root hair elongation, *alf6* mutants showed a pleiotropic phenotype comprising reduced anthocyanin accumulation and altered root architecture in response to low Pi, suggesting that ALF6 is an upstream regulator of several Pi-related processes. Intriguingly, similar to what has been observed on plants with altered *HDA19* abundance, various genes involved in membrane lipid remodeling were among the putative targets of ALF6, suggesting a close connection between membrane lipid remodeling and root hair differentiation. Transcription of both *HDA19* and *ALF6* is not responsive to Pi starvation, indicating that some of the key players escape detection in transcriptional or proteomic surveys. The two studies also indicate that substantial regulation of cellular Pi homeostasis occurs at the histone level. It is tempting to speculate that histone acetylation and methylation interacts to control the transcription and/or post-transcriptional processing of genes that impact the phenotypic readout. Notably, it was reported that H3K4 trimethylation was absent in the *HDA6* mutant *axe1-5,* indicating that HDA activity is required for H3K4 methylation^39^, supporting such a scenario.

### Iron-phosphate interactions may impact intercellular communication

Several lines of evidence have provided support for a role of iron in the attenuation of root hair elongation in response to Pi starvation. Generally, Pi-deficient plants accumulate a surplus of iron despite strongly down-regulated iron acquisition genes^13^,^15^. Notably, restricted elongation of primary roots upon Pi deficiency does not occur in the absence of iron, which led to the assumption that this response does not primarily reflect an acclimation to Pi deficiency but is rather caused by iron toxicity^40^. Recently, this view has been challenged by the observation that a putative ferroxidase, LPR1, and the P5-type ATPase PDR2 are required to allow for meristem-specific iron and callose deposition which in turn regulates symplasmic communication in the RAM and elongation zone during Pi starvation^41^. Several iron-responsive genes are differentially regulated between *HDA19* knock-down and overexpression lines, with higher expression in the HDA19-RNAi line. Assuming that a sophisticated control of iron uptake and transport is required to regulate cell-to-cell signaling in Pi-deficient plants, it appears plausible that the strength of the positional signal that controls epidermal cell fate and longitudinal cell length is modulated in plants with altered HDA19 abundance due to subtle alterations in iron distribution. Growth on low Pi media may further decrease the signal, thereby increasing the probability of hair formation in the N position.

In conclusion, our data highlight a novel non-Pi-responsive modulator of the PSR that controls cell length and, as a consequence, root hair density. In addition, the Pi deficiency-induced expression of key regulatory factors such as the SPX domain-containing transcription factors *SPX3* and *SPX1* is decreased in *HDA19* knock-down lines. It is plausible that such changes are mediated by interactions of histone PTMs and Pi-responsive factors. These bind to histone writers and histone readers that control transcription and fine-tune cell length and gene expression to the availability of Pi.

## Methods

### Plant growth conditions

*Arabidopsis thaliana* (L.) Heynh. plants were grown in a growth chamber on a solidified medium as described by Estelle and Somerville^42^. Seeds of the accession Wassilewskija (Ws-2) and Columbia (Col-0), HDA6-RNAi (CS24038), HDA9-RNAi (CS30879), *had17* (SALK_090088) and *hda18* (SALK_006938) plants were obtained from the Arabidopsis Biological Resource Center (Ohio State University). Plants over-expressing *HDA19* under the control of the 35S promoter (35S:HDA19), HDA19 RNAi plants, *hda19* mutants (*hda19-1*) and HDA6-RNAi plants have been described previously^37^,^43^,^44^. Seeds were surface-sterilized by immersing them in 5% (v/v) NaOCl for 5 min and 70% ethanol for 7 min, followed by four rinses in sterile water. Seeds were placed onto Petri dishes and kept for 1 d at 4 °C in the dark, before the plates were transferred to a growth chamber and grown at 21 °C under continuous illumination (50 μmol m^−2^ s^−1^; Phillips TL lamps). The medium was solidified with 0.4% Gelrite pure (Kelco), the pH was adjusted to 5.5. Low Pi conditions were obtained by growing plants on media containing 2.5 μM KH_2_PO_4_, while the Pi concentration of control medium was 2.5 mM KH_2_PO_4_. The lower concentration of potassium due to the reduced KH_2_PO_4_ concentration was compensated for by the addition of KCl.

### Microarray analysis

The Affymetrix gene chip *Arabidopsis* ATH1 Genome Array was used for microarray analysis. Total RNA sample were prepared as described above. All RNA samples were quality assessed by using the Agilent Bioanalyzer 2100 (Agilent, Santa Clara, USA). Complementary RNA synthesis was performed by use of the GeneChip One-Cycle Target Labeling Kit (Affymetrix, Santa Clara, USA). Hybridization, washing, staining, and scanning procedures were performed as described in the Affymetrix technical manual.

Gene expression data were imported directly into GeneSpring (version 11.5, Agilent, Santa Clara, USA). The software was used to normalize the data per chip to the 50^th^ percentile and per gene to the control samples. Genes that were flagged as absent in two replicates were not considered in the analysis. *P* values for the Benjamini and Hochberg method (false discovery rates; FDRs) were calculated by GeneSpring. Transcripts were defined as differentially expressed that showed delta signal changes larger than the mean expression value of the whole data set or 2-fold changes with *P* < 0.05.

### Real-time RT-PCR

For qRT-PCR, total RNA was extracted from the roots using the RNeasy Plant Mini Kit (Qiagen) and DNase treated with the Turbo DNA-free Kit (Ambion) following the manufacturer’s instructions. cDNA was synthesized using SuperScript III reverse transcription kits (Invitrogen) following the manufacturer’s instructions. Real-time PCR was performed using Power SYBR Green PCR Master Mix (Applied Biosystems) on an Applied Biosystems 7500 Fast Real-Time PCR System with programs recommended by the manufacturer. Samples were normalized first to an endogenous reference (*AtTUA*) and then the relative target gene was determined by performing a comparative ΔΔCt. The following primers were used: *TUA* (At5g19770) fwd: GTGCTGAAGGTGGAGACGAT, rev: AACACGAAGACCGAACGAAT; *SPX1* fwd: GAAGAGCACAATCGCTGCCTT, rev: TGGCTTCTTGCTCCAACAATG; *SPX3* fwd: GCGCCGGTGGAATCTATTTT, rev: GCAACTCCTTGTGGTGGATGA; pri-miR399 fwd: TTACTGGGCGAATACTCCTATGG, rev: ATTTTACTTGCATATCTAGCCAATGC.

### Measurement of root hair length and density

Confocal images with a scale bar of 100  μm at 10 X resolution were used for measuring the root hair length. A ZEISS DISCOVERY V.12 microscope equipped with an ocular scale bar was used for measuring root hair density at 2 to 6 mm from the tip of the primary root. Statistical significant deviations from the wild type were determined by Student’s *t*-test. Micrographs were taken between 0 to 8 mm from the tips of primary roots.

For cross-sections, root samples were fixed, dehydrated and then embedded in Technovit 7100 (Heraeus Kulzer, Wehrheim) resin in gelatin capsules. Transverse sections (30  μm) were cut using a RM 2255 Leica microtome (Leica, Nussloch, Germany). Sections were dried and stained with toluidine blue (0.05%) on glass slides and examined using bright-field on an Imager Z1 microscope (Zeiss, Jena, Germany).

### Confocal microscopy and cell length measurements

Plants were placed in 10 mg/ml propidium iodide solution (PI) for one minute and gently rinsed with water for two minutes. The root was removed and mounted in fresh water. The roots where then observed using a confocal laser scanning microscope (Zeiss LSM510 Meta). The peak excitation λ and emission λ for PI was 536 nm and 620  nm, respectively.

The cell length of trichoblasts and atrichoblasts was measured using ImageJ (http://rsb.info.nih.gov/ij/). The position of each cell was calculated from the cumulative length of all cells between the cell and the quiescent center. The data sets were then smoothed and interpolated into 25-mm-spaced data points using a kernel-smoothing routine^45^, which was performed using a Microsoft Excel macro that enable the average calculation between replicate roots.

### Northern blot analysis

Total RNA was extracted from roots with TRIzol® Reagent (Invitrogen) and 10 μg of total RNA were loaded in each lane of a denaturing 17% polyacrylamide gel, size-separated by electrophoresis, and transferred to a nylon membrane. The following hybridizations were performed as described in Sunkar and Zhu^46^ using^32^ P-labeled probes complementary to miR399d.

### ICP-OES analysis

Mineral nutrient analysis was determined by inductively coupled plasma optical emission spectrometry (ICP-OES) as described in Rodríguez-Celma *et al.*^29^. Five plants were harvested per treatment and genotype.

## Additional Information

**How to cite this article**: Chen, C.-Y. *et al.* The histone deacetylase HDA19 controls root cell elongation and modulates a subset of phosphate starvation responses in *Arabidopsis*. *Sci. Rep.* **5**, 15708; doi: 10.1038/srep15708 (2015).

## Supplementary Material

Supplementary Information
